# Pathogen Reduction Potential in Anaerobic Digestion of Organic Fraction of Municipal Solid Waste and Food Waste

**DOI:** 10.3390/molecules25020275

**Published:** 2020-01-09

**Authors:** Przemysław Seruga, Małgorzata Krzywonos, Zbigniew Paluszak, Agnieszka Urbanowska, Halina Pawlak-Kruczek, Łukasz Niedźwiecki, Hanna Pińkowska

**Affiliations:** 1 Department of Bioprocess Engineering, Wrocław University of Economics and Business, Komandorska 118/120, 53-345 Wrocław, Poland; malgorzata.krzywonos@ue.wroc.pl; 2 Department of Microbiology and Food Technology, University of Technology and Life Science, Bernardyńska 6/8, 85-029 Bydgoszcz, Poland; paluszak@utp.edu.pl; 3 Faculty of Environmental Engineering, Chair in Water and Wastewater Treatment Technology, Wroclaw University of Science and Technology, Wyb. Wyspiańskiego 27, 50-370 Wrocław, Poland; agnieszka.urbanowska@pwr.edu.pl; 4 Department of Boilers, Combustion and Energy Processes, Faculty of Mechanical and Power Engineering, Wroclaw University of Science and Technology, Wyb. Wyspiańskiego 27, 50-370 Wrocław, Poland; halina.pawlak@pwr.edu.pl (H.P.-K.); lukasz.niedzwiecki@pwr.edu.pl (Ł.N.); 5 Department of Industrial Chemistry, Wrocław University of Economics and Business, Komandorska 118/120, 53-345 Wrocław, Poland; hanna.pinkowska@ue.wroc.pl

**Keywords:** *Ascaris suum*, *Salmonella*, *Enterococcus*, biowaste, fermentation, hygienization

## Abstract

Anaerobic digestion (AD) is a commonly used method of processing waste. Regardless of the type of the used digestate (fertilizer, feedstock in case of solid-state fermentation, raw-material in case of thermal treatment) effective pathogen risk elimination, even in the case of high pathogen concentration is essential. An investigation of the survival time and inactivation rate of the *Salmonella Senftenberg W*_775_, *Enterococcus* spp., and *Ascaris suum* eggs during thermophilic anaerobic digestion performed on laboratory scale and confirmation of hygienization in full-scale operation were performed in this study. Except for sanitization efficiency, the AD process performance and stability were also verified based on determination of pH value, dry matter content, acidity, alkalinity, and content of fatty acids. The elimination of pathogen was met within 6.06 h, 5.5 h, and about 10 h for the *Salmonella Senftenberg W*_775_, *Enterococcus* spp., and *Ascaris suum*, respectively in the laboratory trials. The obtained results were confirmed in full-scale tests, using 1500 m^3^ Kompogas^®^ reactors, operating in MBT Plant located in Poland. Sanitization of the digestate was achieved. Furthermore, the process was stable. The pH value, suspended solids, and ammonium content remained stable at 8.5, 35%, and 3.8 g/kg, respectively. The acetic acid content was noted between almost 0.8 and over 1.1 g/kg, while the concentration of propionic acid was noted at maximum level of about 100 mg/kg. The AD conditions could positively affect the pathogen elimination. Based on these results it can be found that anaerobic digestion under thermophilic conditions results in high sanitation efficiency.

## 1. Introduction

According to the EU regulation [1] food and kitchen waste belong to category 3 and require proper management to eliminate the potential health risks. Special treatment parameters ought to be maintained. Regarding to a biogas plant, at the initial stage, kitchen waste has to be pasteurized within a minimum of 60 min in a minimum temperature at 70 °C. Additionally, waste has to be macerated to a particular size below 12 mm. Furthermore, the presence of some pathogens (*Salmonella* spp., *E. coli* or *Enterococcus* spp., and *Ascaris* spp.) has to be monitored.

*Salmonella* is one of the most likely pathogens to be spread in the environment via animal slurry and sewage sludge [2]. All serotype of *Salmonella* are potentially pathogenic to both animals and people. Foodborne enteritis (food poisoning) is the most common infection caused by *Salmonella*, which is a significant public health concern. Salmonellosis is the second most commonly reported gastrointestinal infection and an important cause of foodborne outbreaks in the European Union [3]. *Salmonella* may survive for a considerable length of time, for example in slurry for more than 77 days with growth in temperature ranging from 6 to 47 °C [4].

Verotoxin-producing *E. coli* O157 is an important foodborne pathogen that causes colitis or hemolytic uremic syndrome in people [5]. The bacteria are found in bovine manure. Wang et al. [6] report that *E. coli* O157:H7 survives for up to 10 weeks and can produce verotoxins for up to 10 weeks.

Ascariasis is caused by infection with the giant roundworm *Ascaris* lumbricoides, with around 760 million cases worldwide. Although infections are particularly common in developing countries where sanitation and hygiene is poor, ascariasis exhibits a cosmopolitan distribution, with cases also described in developed countries. It was recently estimated that ascariasis contributes 1.31 million disability-adjusted life years to the global burden of disease. The closely related parasite *Ascaris suum* infects innumerable pigs across the globe and is especially common in organic and extensive farming systems Infections in pigs are associated with production losses owing to reduced growth and low feed conversion efficiency, with livers unfit for human consumption [7].

The extreme fertility of the *Ascaris suum* and the very high resistance of the eggs cause a considerable risk of environmental pollution for a long period of time [8]. On average a mature female produces about 200,000 eggs, and in some instances, up to 1,600,000 eggs during one day [9]. It was estimated that the eggs survive in soil fertilized with sewage not subjected to prior sanitization for even up to 10 years [10].

The anaerobic digestion (AD) of the organic waste has been used widely as a form of energy recovery [10], which fits with the actual tendency to avoid, reduce, reuse, recycle, recover, and treat waste to minimize the disposal and focus on the circular economy [11]. It can be found that AD is the most promising and sustainable process for treatment of organic waste while it is also an environmentally friendly method [12], which can ensure proper hygienization. However, the advantages of AD can be negated due to improper management of the remaining digestate. The recontamination may occur if storage conditions favor regrowth of microorganism. After sanitization of manure using AD (elimination of *E. coli* and *Salmonella*), control levels of all potential pathogens were recovered after 3 months, which could be considered as a safe delay between land spreading and harvesting [13]. Additionally, separation of fresh and digested waste might help in recontamination prevention.

In full-scale biogas plants a wide variety of biowaste is used to produce the biogas. All of the biowaste, i.e., manure, biological household waste, food waste, sewage sludge can contain pathogens causing infections in humans and animals [14,15]. The digested residue might be used as a fertilizer and must therefore be hygienically safe. Pathogens are rapidly inactivated by heat; the combination of short time and high temperature (>7 min at 70 °C) or long time and low temperature (>3 days at 55 °C) were reported to be effective in pathogen reduction under laboratory conditions [16]. The minor factors affecting pathogen decay or loss of viability during AD are the hydraulic retention time (HRT), temperature, present volatile fatty acids (VFA), batch or continuous digestion, as well as bacterial species and available nutrients [14] In general AD reduces pathogen content, especially when thermophile conditions are adopted [17]. The mesophilic process has been reported to be less effective in reducing pathogens because of using temperature that are lower than temperatures used by the thermophilic process, although pathogen reduction can also be achieved by means of this process [18].

Nearly 95% of the digestate produced in Europe can be used as an organic fertilizer to replace chemically produced fertilizers [19]. However, fertilization using this type of fertilizer is currently limited due to its quality, nutrient content, possible land contamination, sanitary safety, or even lack of fertile land [19].

Due to the emerging limits of using digestates the fertilizer, a novel approach which uses digestate as a feedstock for further biotransformation through solid-state fermentation (SSF) is currently under consideration [20]. SSF can be an attractive technology. It allows the use of solid substrates for biotransformation into value-added bioproducts, such as: Hydrolytic enzymes, biosurfactants, aromas, biopesticides, or bioplastics [20]. Moreover, application of torrefaction or the pyrolysis for recycling biowaste formed during AD, is currently considered as another digestate management path [21].

Regardless of the intended use of the digestate (fertilizer, feedstock for SSF, raw-material, for thermal treatment) this type of waste represents a great environmental hazard, as it is a transmission source of pathogens of infectious and invasive diseases and a favorable environment for the incubation and long-term survival of pathogenic microflora [21,22]. Thus, effective elimination of pathogen risk, even in the case of high pathogen concentration is essential. Although the thermal inactivation of pathogens (*Salmonella* spp., *E. coli* or *Enterococcus* spp., and *Ascaris* spp.) has been widely studied in laboratory conditions [16,17], attention must be paid when applying the results as well as time–temperature relationship analyzed in laboratory to large-scale systems [23]. Here, exposure times required for inactivation of pathogens may be affected by uneven heating, fluctuating temperatures or shielding properties of solids [23,24]. Thus, the transfer of the laboratory results to the full scale systems requires further investigation and is an interesting area of research.

The aim of this study was to investigate the survival time and inactivation rate of the *Salmonella Senftenberg W*_775_, *Enterococcus* spp., and *Ascaris suum* eggs introduced into the biomass of organic waste, without pasteurization, and subjected to thermophilic anaerobic digestion at laboratory scale as well as to confirm hygienization effect in full-scale operation to ensure digestate’s sanitary safety.

## 2. Results and Discussion

According to EU regulation [1] food and kitchen waste belong to category 3 and require pasteurization to eliminate the potential health risks. The anaerobic digestion under thermophilic conditions has been determined to be a suitable process for eliminating pathogenic microorganism, instead of pasteurization [17].

Results of the conducted laboratory research showed elimination of *Salmonella Senftenberg W*_775_ and *Enterococcus* spp., bacteria along with inactivation of the *Ascaris suum* eggs, during the co-digestion of OFMWSW with 10% food waste addition. During the anaerobic digestion the temperature inside of the laboratory bioreactor was maintained at 54 ± 0.3 °C level. The marked colony formed units (CFU) for both bacterial species during the experiments are shown in Table 1.

**Figure 1:**
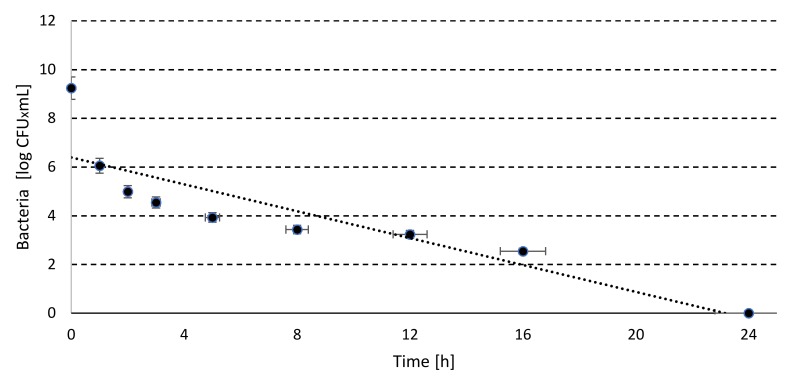
The regression line for change of kinetics of *Salmonella* population in laboratory scale.

Based on this data and the trajectory of the regression line (Figure 1) it can be indicated that the required by the EU regulation [1] *Salmonella* spp. elimination by 5 order was obtained after 6.06 h.

The total survival time of the kinetics change *Salmonella Senftenberg W*_775_ reached 9.93 h (Table 2), which corresponded with the elimination rate of 0.53 log CFU within one hour (Table 2). The *Salmonella* counts decreased since the beginning of the process (Figure 1), while Jiang et al. [3] reported increment in *Salmonella* counts, of both high and low inoculation. The plentiful initial supply of nutrients and low volatile fatty acids content could be the cause of such results. Nevertheless, *Salmonella* was completely eliminated within 6–7 days [3].

Temperature is the most important factor pertaining to the survival of pathogenic bacteria during AD [25]. Digestion taking place at higher temperatures requires less time for bacterial inactivation and this causes bacteria to die much faster under thermophilic rather than mesophilic digestion [26]. It was already demonstrated by Olsen and Larsen [27] in a study including *Salmonella typhimurium*, *Salmonella dublin*, *E. coli*, *Staphylococcus aureus*, *Streptococcus faecalis*, *Erysopelotrix rhusiopathiae*, *Bacillus cereus*, and *Clostridium perfringens*. *Salmonella*, and *M. paratuberculosis* which were inactivated within 24 h under thermophilic anaerobic digestion compared to weeks and even months under mesophilic anaerobic digestion.

Full inactivation of the *Ascaris suum* eggs was obtained after 12 h of laboratory experiments (Table 3). The total time of survival ranged from 9.84 to 10.0 h (Table 4), which corresponded with a decrease in the percentage of live eggs, by 7.11 and 6.7 during one hour (Table 4), respectively for each bioreactor.

The eggs of *A. suum* are rather resistant to environmental conditions, that is why some sanitization methods appear to be ineffective and eggs can maintain their infectious properties even for 20 months up to 6 years [28]. In the present study, the *A. suum* eggs were introduced into the biomass and subjected to AD under thermophilic conditions. Papajova et al. [29] stated that exposing *A. suum* eggs to the thermophilic conditions is the best way of elimination, due to inactivation of the enzymatic system of the egg. Harrof et al. [23] investigated the time–temperature relationship for the inactivation of *Ascaris* eggs under mesophilic temperatures in aerobic and anaerobic treatment. They observed inactivation in AD at temperatures between 37 and 45 °C, while at lower temperatures *Ascaris* stayed active. The time required for a 3-log reduction in *Ascaris’* viability decreased from 57.5 to 2.12 days as the temperature increased [23]. Ligocka and Paluszak [30] who compared composting and fermentation under mesophilic conditions have also confirmed that the fastest inactivation during AD is taking place at 55 °C. A decrease in the percentage of invasive eggs amounted to almost 8% per hour [30], which is similar to a decrease obtained in present research (6.7–7.1%/h).

The full-scale research was conducted in anaerobic digestion plant using the OFMSW with about 5% addition of restaurant waste as co-substrate in the course of 2 months. Addition of food waste was set up and limited based on the previous research results [31].

**Figure 2:**
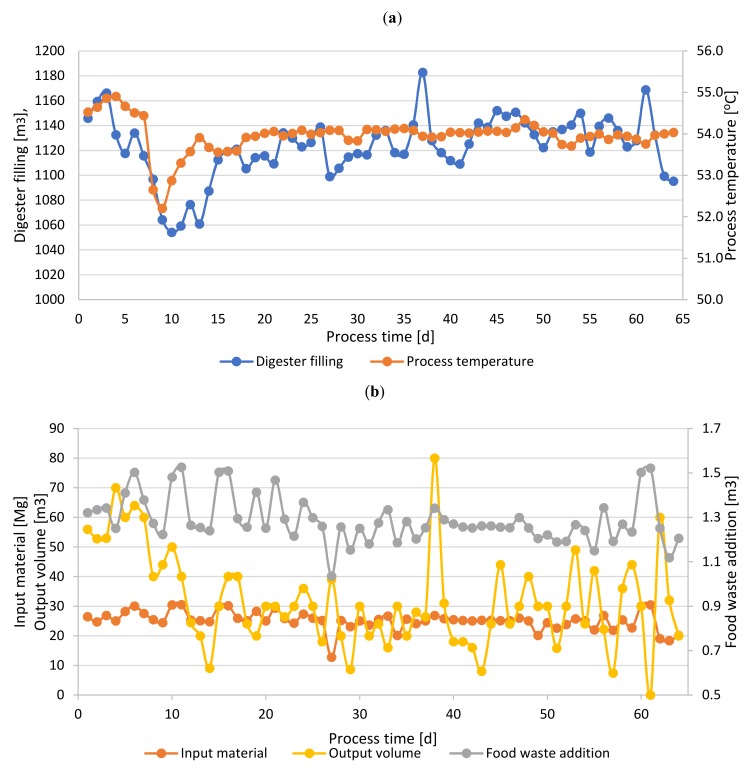
Changes of the anaerobic digestion parameters: (**a**) digester filling and process temperature and (**b**) material flow: Input, output, and food waste streams.

The average daily load of 25.2 ton of the OFMSW was supplied with 1.29 m^3^ food waste addition (Figure 2). The filling of the digester was maintained at the level of approximately 1120 m^3^, by means of adjusting the extraction volume to the input stream (Figure 2). The digester’s heating installation was set to ensure thermophilic conditions (Figure 2). The recorded hydraulic retention time (HRT) amounted to 42.5 days. After the required retention time samples of digestate were collected and presence of bacteria (*Salmonella* spp. and *Escherichia coli*) was measured. Neither *Salmonella* spp. nor *E. coli* was detected in any of the five examined samples of the digestate.

The pH value, volatile fatty acids, and ammonia concentrations were found to be significant to the *Salmonella* elimination during AD [3]. Moreover, these factors impact the stability of the process. Regarding to the full scale research, not only sanitization efficiency but also process stability is important as it ensures the continuity of exploitation.

**Figure 3:**
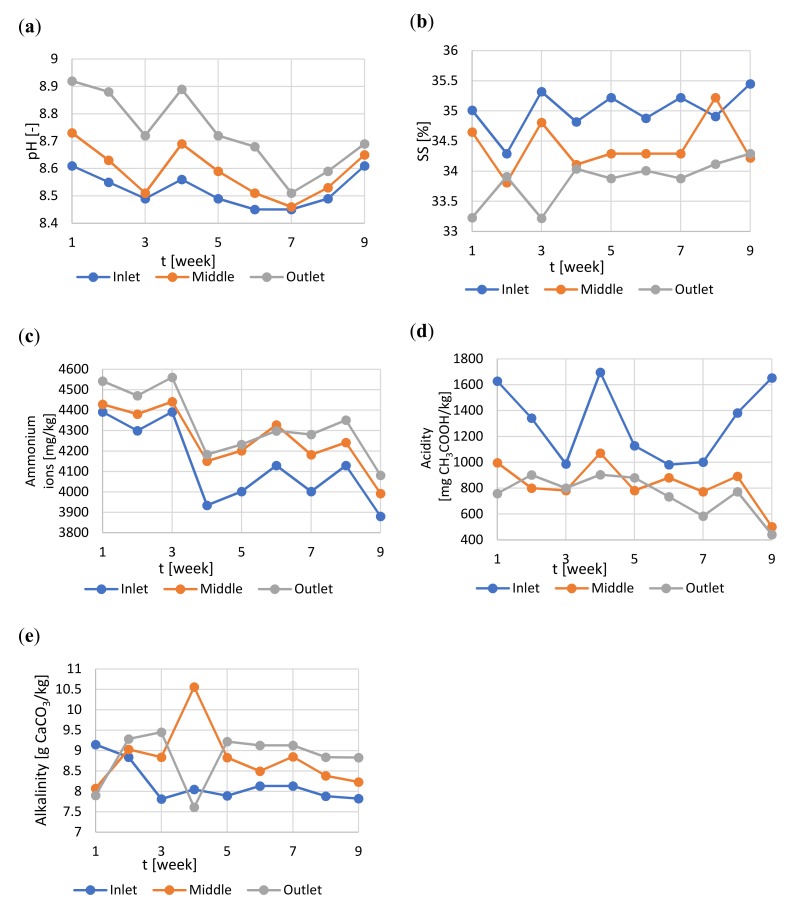
Changes of the parameters during the anaerobic digestion process in full scale: (**a**) pH value; (**b**) suspended solids; (**c**) ammonium ions concentration; (**d**) acidity; (**e**) alkalinity.

The pH value is considered to be the anaerobic digestion process stability factor [32]. Normally, it should be maintained between 7.2 and 7.8. The pH-value decreases are usually caused by organic overloading of the digester [32]. The increase in alkalinity (pH values above 8.0) impacts the NH_3_ and NH_4_^+^ dissociation balance. However, mainly due to the buffering capacity of the digester, pH changes are usually only detectable when the process is already unstable. Therefore, it can be found that the determination of the low-fatty acids (acetic, propionic, butyric, and valeric) concentrations, with their ratios, provides the most reliable information regarding to the stability of digestion. Regarding to the sanitization aspect, the highest growth of *Salmonella* was observed at pH 7.0 [3]. No significant differences were observed between pH 7.0 and 8.0, while at pH 9.0 and 6.0 bacterial growth was significantly lower [3], indicating that both acid and alkaline conditions inhibit the growth of *Salmonella*. In our study the pH value remained stable at 8.5 in the digester inlet section, with typical increment towards the outlet (Figure 3a) and suggested process stability and bacterial suppression.

**Figure 4:**
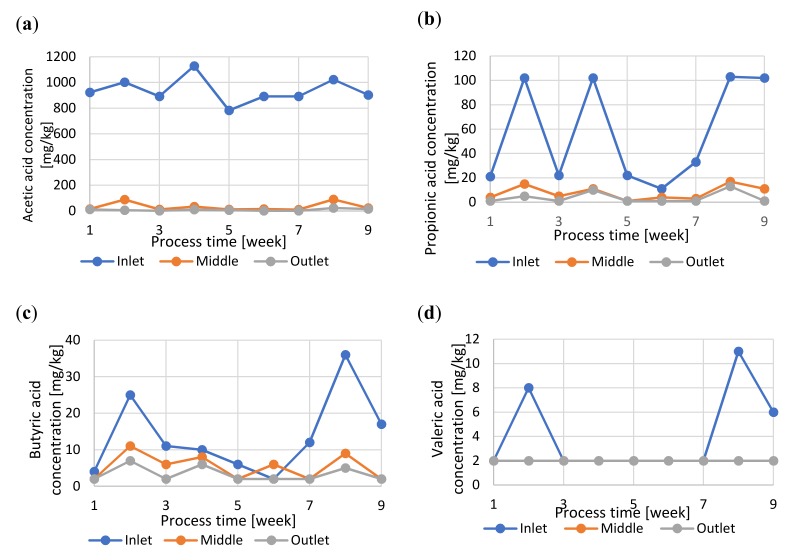
The changes in fatty acids concentrations during the anaerobic digestion in full-scale: (**a**) acetic acid; (**b**) propionic acid; (**c**) butyric acid; (**d**) valeric acid.

Taking into consideration the other weekly verifications of process parameters performed during anaerobic digestion it can be concluded that the process was stable (Figure 3 and Figure 4). The suspended solids were at the 35% level in the feeding section, and decreased to about 33.8% towards the extraction section (Figure 3b). The concentration of ammonium ions have also remained stable (Figure 3c) at over 3.8 g/kg, which could positively affect sanitization. Jiang et al. [3] concluded that the reduction of *Salmonella* was increasing slowly along with the increase in total ammonia concentration. The increase in ammonia concentration from 50 to 500 mmol/L caused 25.0% to 65.4% increase in the bacterial reductions. However, they have also observed that the inhibitory effect of ammonia was much lower than the effect caused by similar VFA concentrations (40.6–86.1%) [3].

In the full-scale research the acetic acid content ranged from almost 0.8 to over 1.1 g/kg, with decrease in the concentration to few mg/kg at the end of the digester (Figure 4a). The concentration of propionic acid was noted at maximum level of about 100 mg/kg in the inlet section and at about 10 mg/kg in the outlet section of the digester (Figure 4b). The concentrations of the remaining acids (butyric, and valeric) did not exceed 5 mg/kg in the outlet section of the digester (Figure 4c,d), which could also favorably impact sanitization.

Whether the reactor is stable can be also deduced from acidity, alkalinity and its ratio. When the ratio is below 0.3 the process has a robust stability, while when the ratio is above 0.5 the risk of acidification and process breakdown appears [33]. In the conducted research the ratio did not exceed 0.2 (Figure 3d,e), which proves process stability.

Regarding to the *Salmonella* reduction it can be assumed that it increases along with the increment of VFA concentration. High VFA concentrations meant highly available acid molecules and a high osmotic gradient across cell membranes, resulting in increased toxicity to *Salmonella* [3]. The authors of the research came up with the inhibitory sequence of volatile fatty acids valeric (HV) > isovaleric (HIV) ≥ propionic (HPR) ≥ mixed volatile fatty acid (MV-FA)() > butyric (HB) ≥ isobutyric (HIB) > acetic (HAC). Also, Back et al. [34] pointed to the HPR > HAC > formic acid sequence in reference to inhibitory effects against multiplication of *Enterobecter sakazakii.* However, Salsi et al. [35] reported the following sequence acetic acid (HAC) > propionic acid (HPR) > butyric acid (HB) during *Salmonella* inactivation. Thus, the inhibitory effect of HAC on *Salmonella* may not be clear due to its short chain. On the other hand it can be assumed that the inhibitory effect of linear chain VFA might be greater than that of the branched chain, because linear chain VFA could cross cell’s membrane more readily [3]. This statement might be confirmed by the results of our research, considering good sanitization effect and high HAC concertation when compared to other VFA concentrations. However, detailed inactivation mechanism requires to be studied further.

**Figure 5:**
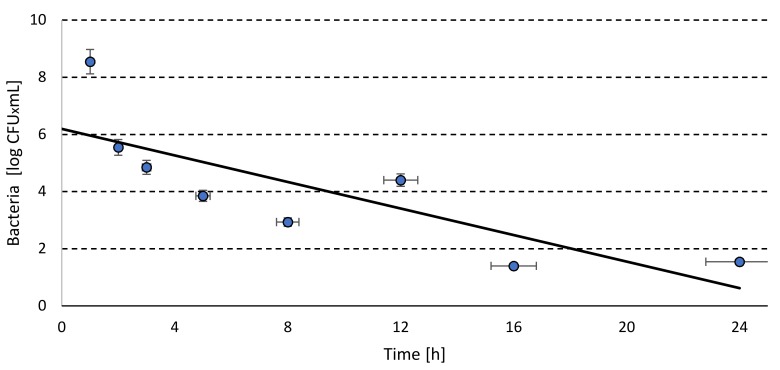
The regression line for kinetics change of the *Enterococcus* population in laboratory scale.

Regarding to the *Enterococcus* spp. the elimination required by law regulation was observed in laboratory run within 5.5 h (Table 1, Figure 5). The total time of the *Enterococcus* spp. survival reached 8.29 (Table 2), which corresponded with the elimination rate of 0.91 logCFU during one hour (Table 2).

Dennehy et al. [36] examined the impact of the composition of feedstock (pig manure (PM) and food waste (FW)) as well as of the impact of HRT on *E. coli* and *Enterococcus* spp. elimination during anaerobic digestion at 39 °C in 10 L bioreactor. The authors concluded that the composition of feedstock (PM/FW mixing ratio) had no clear effect on the observed concentrations of any of the bacteria present in the digestate. Additionally, no consistent trend between reactors was observed as the HRT has been increased from 29 to 41 days. The decrease in *Enterococcus* was observed, while *E. coli* concentrations appeared to increase just slightly [36]. Similar findings might be formulated on the basis of present research results comparing laboratory and full-scale experiments. In both experiments, despite the addition of different restaurant waste and HRT pathogen the elimination was noticed. Limited organic load ratios used to ensure process stability, high HRT investigated, and the high level of stability observed throughout this present study could explain why no differences were observed [36]. Explanation as to why a high level of sanitation was achieved may also be down to the mechanism responsible for pathogen inactivation (high free ammonia nitrogen, high volatile fatty acids, competition from other microbial species, and resource limitations) [37].

Taking into consideration the high level of sanitation after anaerobic digestion under thermophilic conditions, the process can effectively substitute high temperature treatment, providing pasteurization efficiency. *E. coli* and *Salmonella* phage 28B, as well as *A. suum* eggs were found to be fully eliminated after 30 min of treatment at 70 °C [14]. However, there is no need to waste energy pasteurization thanks to the thermophilic AD sanitation efficiency. AD reduces the abundance of pathogens, contributing to more hygienic crops. Pre-sanitation is also important when the digestate is used as a feedstock for further biotransformation through SSF [20], torrefaction, or pyrolysis [21].

## 3. Materials and Methods

### 3.1. The Laboratory Research

The laboratory research consisted of a batch experiment, using a bioreactor (type WP-16453) with two digesters (total volume 35 L), mechanical shaft and a heating system. Each digester was filled with 30 L of digested organic fraction of municipal solid waste (OFMSW) collected from the mechanical-biological treatment plant (MBT) located in Poland. Digesters were kept in 54 °C. The suspension of the *Salmonella Senftenberg W*_775_ H_2_S, *Enterococcus* spp. (10 mL per liter of bioreactor filling) and the eggs of *Ascaris suum* which were placed inside the perlon carriers were introduced. The samples were collected after: 0; 1; 2; 3; 5; 8; 12; 16; and 24 h.

### 3.2. The Suspension of A. suum

Eggs of the *Ascaris suum* were isolated from sexually-mature female individuals obtained from meat processing plants. The eggs were mixed with water and 1mL of the suspension (density of 1–6 thousand eggs/mL) was introduced into the perlon sacks, with a 28 μm mesh where it was subjected to the physio-chemical factors generated during the processes. The perlon sack prevented the eggs from getting out of the biomass.

### 3.3. The Bacterial Suspension

The volume of seeding totaled 0.1 mL of thawed suspension of bacterial strain. The bacteria were inoculated under aseptic conditions on the Petri dishes with agar. The bacterial suspension was adjusted spectrophotometrically to 4.5 × 10^9^ per mL at 550 nm.

### 3.4. The Parasitological Determination

At the right time, the sacks were removed from the apparatuses and their content was transferred to the Petri dishes. Incubation of the eggs was conducted for 28 d at 28 °C. Subsequently, the percentage of live larvae was calculated using an optical microscope (300×) (DELTAOptical, Nowe Osiny, Poland). The control was a suspension of eggs subjected to incubation directly after being collected from the uterus of a sexually-mature individual.

### 3.5. The Microbiological Determination

Isolation of the *Salmonella Senftenberg W*_775_ was performed using: 1% peptone water (24 h at 37 °C); Rappaport–Vassiliadis culture medium (24 h at 42 °C) and brilliant green, phenol red, lactose agar (BPL) (24 h at 37 °C).

The *Enterococcus* spp. isolation was performed using: broth with azide and glucose (24–48 h at 37 °C); agar with kanamycin, esculin, and azide (24–48 h at 37 °C). Final identification was carried out based on the Phadabact D-Strep Test.

### 3.6. The Statistical Analysis

Simple linear regression analysis was applied to calculate both the total time of survival of the microorganisms and the fall in percentage. Furthermore, theoretical survival time, elimination rate and decimal elimination time (DET) were calculated.

### 3.7. The Full-Scale Research

The research was conducted at the MBT Plant, located in the Lower Silesia a region, in southwestern Poland. The anaerobic digestion (AD) processes were carried out in a 1500 m^3^, full-scale Kompogas^®^ reactors (ZGO Gać, Oława, Poland) with the OFMSW and food waste (restaurant waste) addition used as feedstock. Food waste was macerated to size below 12 mm. The temperature was maintained at 54 °C. The agitation speed was set at 0.45 rpm, in 300 s operation and 120 s break cycles, with alternating directions. The operating volume in the digester was determined by means of setting up the extraction pump operation time. Process parameters were archived daily by the central control system. At the end of the experiment five samples of digestate were obtained and the quantity of bacteria was determined. Furthermore, every week, samples from the digester inlet, outlet and middle section were collected. To verify performance and stability of the process the pH value, dry matter content, acidity, alkalinity and content of fatty acids (acetic (HAC), propionic (HPR), butyric (HB) with isobutyric (HIB), and valeric (HV) with isovaleric (HIV)) were determined.

### 3.8. Analytical Methods

The content of fatty acids was determined by means of gas chromatography (Varian GC 450) (Varian BV, Middelburg, The Nederlands) with a FID detector (H_2_: 30 mL/min, air: 300 mL/min, He: 30 mL/min) (Varian BV, Middelburg, The Nederlands). Helium (constant flow through the 1 mL/min column) was used as the carrier gas with 1:30 split.

The acidity and alkalinity were determined by pH-metric titration, in compliance with the standard methods [25]. The acidity/alkalinity ratio (index R) was also determined.

The suspended solids (SS), dry organic mass, and pH were determined as per the standard methods [38].

## 4. Conclusions

This study assessed the effects of pathogen removal during the anaerobic co-digestion of food (restaurant waste) under thermophilic conditions and confirmed high sanitation effect. The laboratory and full-scale experiments confirm the elimination of *Salmonella Senftenberg W*_775_ and *Enterococcus* spp. bacteria along with inactivation of the *Ascaris suum* eggs, during co-digestion of OFMWSW with addition of restaurant waste.

Elimination of the *E. coli*, *Enterococcus*, *Salmonella*, and the *A. suum* eggs was proven in laboratory and digestate sanitization was confirmed in full-scale experiments. Even in the case of strongly contaminated feedstock, the anaerobic digestion in thermophilic conditions may guarantee a product that is microbiologically safe for the environment. The requirements of law regulation were met within 6.06 h, 5.5 h, and about 10 h for *Salmonella Senftenberg W*_775_, *Enterococcus* spp., and *Ascaris suum*, respectively. This rapid inactivation could prevent environmental pathogen contamination after application of digestate.

## Figures and Tables

**Table 1:** Colony formed units (CFU) of analyzed bacteria in laboratory scale.

Bacteria	Time [h]	Run I [CFU]	Run II [CFU]
***Salmonella Senftenberg W*_775_**	0.0	9.5 × 10^8^	2.5 × 10^9^
	1.0	15.0 × 10^5^	7.5 × 10^5^
	2.0	15.0 × 10^4^	4.5 × 10^4^
	3.0	2.5 × 10^4^	4.5 × 10^4^
	5.0	9.5 × 10^3^	7.5 × 10^3^
	8.0	4.5 × 10^3^	9.5 × 10^2^
	12.0	2.5 × 10^3^	9.5 × 10^2^
	16.0	2.5 × 10^2^	4.5 × 10^2^
	24.0	n.d.	n.d.
***Enterococcus***	0.0	0.9 × 10^1^	2.5 × 10^1^
	1.0	4.5 × 10^8^	2.5 × 10^8^
	2.0	4.5 × 10^5^	2.5 × 10^5^
	3.0	9.5 × 10^4^	4.5 × 10^4^
	5.0	4.5 × 10^3^	9.5 × 10^3^
	8.0	9.5 × 10^2^	7.5 × 10^2^
	12.0	2.5 × 10^4^	2.5 × 10^4^
	16.0	2.5 × 10^1^	2.5 × 10^1^
	24.0	4.5 × 10^1^	2.5 × 10^1^

**Table 2:** Statistical parameters of inactivation of bacterial kinetics in laboratory scale.

Bacteria	Linear Regression Equation	Total Survival Time [h]	Elimination Rate [logCFU/h]	DET [h]
***Salmonella Senftenberg W*_775_**	Y = −0.8254*x* + 8.1948	9.93	0.53	1.21
***Enterococcus***	Y = − 0.9089*x* + 8.2036	8.29	0.91	1.91

**Table 3:** The percentage of invasive eggs of the *A. suum* in laboratory scale.

Microorganism	Time [h]	Run I	Run II
***Ascaris suum***	0.0	83%	81%
	0.5	75%	71%
	1.0	75%	69%
	2.0	55%	51%
	2.5	30%	28%
	6.0	5%	8%
	12.0	0%	0%
	24.0	0%	0%
	48.0	0%	0%

**Table 4:** Statistical parameters of the *A. suum* inactivation kinetics in laboratory scale.

Microorganism	Linear Regression Equation	Survival Total Time [h]	Elimination Rate [%/h]	DET [h]
*Ascaris suum—I*	Y = −7.1073*x* + 69.939	9.84	7.11	8.86
*Ascaris suum—II*	Y = −6.6978*x* + 66.964	10.00	6.70	9.00
